# Meta-Analysis of Esophageal Cancer Transcriptomes Using Independent Component Analysis

**DOI:** 10.3389/fgene.2021.683632

**Published:** 2021-10-21

**Authors:** Ainur Ashenova, Asset Daniyarov, Askhat Molkenov, Aigul Sharip, Andrei Zinovyev, Ulykbek Kairov

**Affiliations:** ^1^ Laboratory of Bioinformatics and Systems Biology, National Laboratory Astana, Center for Life Sciences, Nazarbayev University, Nur-Sultan, Kazakhstan; ^2^ Department of Biology, School of Sciences and Humanities, Nazarbayev University, Nur-Sultan, Kazakhstan; ^3^ Institut Curie, PSL Research University, INSERM U900, Paris, France; ^4^ Laboratory of Advanced Methods for High-dimensional Data Analysis, Lobachevsky University, Nizhny Novgorod, Russia

**Keywords:** transcriptomics, genomics, meta-analysis, esophageal cancer, independent component analysis

## Abstract

Independent Component Analysis is a matrix factorization method for data dimension reduction. ICA has been widely applied for the analysis of transcriptomic data for blind separation of biological, environmental, and technical factors affecting gene expression. The study aimed to analyze the publicly available esophageal cancer data using the ICA for identification and comprehensive analysis of reproducible signaling pathways and molecular signatures involved in this cancer type. In this study, four independent esophageal cancer transcriptomic datasets from GEO databases were used. A bioinformatics tool « BiODICA—Independent Component Analysis of Big Omics Data» was applied to compute independent components (ICs). Gene Set Enrichment Analysis (GSEA) and ToppGene uncovered the most significantly enriched pathways. Construction and visualization of gene networks and graphs were performed using the Cytoscape, and HPRD database. The correlation graph between decompositions into 30 ICs was built with absolute correlation values exceeding 0.3. Clusters of components—pseudocliques were observed in the structure of the correlation graph. The top 1,000 most contributing genes of each ICs in the pseudocliques were mapped to the PPI network to construct associated signaling pathways. Some cliques were composed of densely interconnected nodes and included components common to most cancer types (such as cell cycle and extracellular matrix signals), while others were specific to EC. The results of this investigation may reveal potential biomarkers of esophageal carcinogenesis, functional subsystems dysregulated in the tumor cells, and be helpful in predicting the early development of a tumor.

## Introduction

Investigation of cancer profiles is one of the largest sources of genomic and transcriptomic research data. Data has been continuously generated and collected with the advancement of data computing methods and information technology. For instance, a publicly available repository, The Cancer Genome Atlas (TCGA), describes 33 different tumor types, including 10 rare cancers based on both cancerous and normal tissue sets collected from 11,000 patients. The largest fully public gene expression resource is the Gene Expression Omnibus (GEO) database, containing data from 20,000 studies with 500,000 samples, representing over 33 billion individual measurements by November of 2010 (Barrett et al., 2011). While databases represent significant resources for a vast amount of cancer genomics studies, complex issues and challenges remain in obtaining the maximum of useful for understanding cancer biology and use in clinics information from these data (Sudhagar et al., 2018). Available statistical methods work well with data in cases where a large number of observations are available for a small number of variables. However, methods of analysis that are existing today, especially in genomic studies, generate an excessively large number of different variables. In such cases, “unsupervised learning” methods are used utilizing the technique of reducing the dimensionality of data to reduce the multidimensionality of transcriptome data and highlight significant patterns of expression.

Currently, several methodologies for unsupervised data decomposition are widely applied to biological and medical data including Independent Component Analysis (ICA), Principal Component Analysis (PCA), Non-negative Matrix Factorization (NMF). One of the promising and applied mathematical methods for analyzing large data is ICA. There are several studies were conducted in order to assess the performance differences of ICA and PCA (Lee and Batzoglou, 2003; Carpentier et al., 2004; Riva et al., 2005; Mutihac and Mutihac, 2007; Tan et al., 2014; Kairov et al., 2017; Sompairac et al., 2019). A significant proportion of these studies reported the performance stability and reproducibility of the results obtained by multiple runs of ICA. For example, there was a validation of the outperformance of ICA over PCA using regulatory element and phenotype-pathway databases. Moreover, the application of ICA by statisticians for electroencephalograms (EEG) analysis revealed that a more useful data representation was obtained by ICA-based methodology compared to PCA (Bugli and Lambert, 2007). During the application of ICA and PCA algorithms to the same data, it was proved that PCA cannot be used for the extraction of signals with low magnitude and capture only major signals (Sun et al., 2015).

Esophageal cancer (EC) is the eighth most common malignant tumor worldwide accounting for one in every 18 cancer deaths in 2020 (Sung et al., 2021). The two most common histological subtypes are differentiated among EC patients: esophageal squamous cell carcinoma (ESCC) which accounts for about 90% of all incidents and adenocarcinoma (AC). Low survival and high mortality rates for EC are due to the asymptomatic onset of the disease. The 5-years relative survival rate for patients with esophageal squamous cell carcinoma was less than 20% (Abnet et al., 2018). The main symptoms of EC are dysphagia, chest pain, and weight loss occur more often in stage II of the disease. Investigation of the molecular mechanisms of carcinogenesis will allow us to better understand the causes and the triggering mechanisms for the development of the tumor. On the other hand, the identification of genes involved in carcinogenesis will make it possible to identify promising genetic markers for early diagnosis and course prediction of diseases. During the development of EC, cells acquire characteristics of self-sufficiency for growth, evading apoptosis, uncontrolled proliferation, promotion of angiogenesis, invasion of underlined epithelial tissue, and initiation of metastasis. These transformations are characterized by pathologic and homeostatic changes in histological structure, immunological response, as well as the formation of the tumor microenvironment which are reflected in the genomic, transcriptomic, proteomic, and metabolomic levels. Therefore, potential biomarkers from these sources can be obtained for the early diagnosis of cancer.

The ICA-based methodology was suggested to apply for the prediction of tumor subtypes and describe the tumor-related changes using data of gene expression profiles from different data types (single-cell data, RNA-seq data, transcriptomes, and methylomes) (Zhang et al., 2005; Frigyesi et al., 2006; Huang and Zheng, 2006; Zinovyev et al., 2013; Pham et al., 2014; Nascimento et al., 2017; Nazarov et al., 2019). Moreover, ICA was extended to be applied to proteogenomic data of human breast cancer (Teschendorff et al., 2007; Czerwinska et al., 2018; Zhou and Altman, 2018). The results of a recently published article showed the potential of ICA in the identification of pathway-level mechanisms of cancer development (Liu W. et al., 2019). The biological meaning of computed components and their contribution to tumor development were assessed during the analysis of 198 bladder cancer transcriptomes with ICA (Biton et al., 2014). Another study conducted by Cantini et al. showed the application of ICA to colorectal cancer (CRC) which resulted in the cancer-specific and cancer-shared components of CRC subtypes (Cantini et al., 2019).

The aim of this research is to search for potential genetic biomarkers and pathways for early diagnosis of EC using ICA for transcriptomic datasets using the ICA-based deconvolution method. From a fundamental point of view, the results of this proposed investigation may reveal potential biomarkers of tumor processes, functional subsystems dysregulated in the tumor cells, and be helpful in predicting the early development of a tumor process.

## Materials and Methods

### Experimental Data

The four gene expression datasets GSE26886, GSE69925, GSE32701, and GSE21293 were downloaded from the Gene Expression Omnibus (GEO) database (https://www.ncbi.nlm.nih.gov/geo/) that were processed on the Affymetrix HG-U133 Plus 2.0 platform (GPL570). Further information on the sample size and the microarray platforms used for the creation of these datasets is presented in Table 1.

**TABLE 1:** Clinical characteristics of metabolic syndrome-related traits.

Dataset_ID	Types of tissue and samples	Total # of samples	# Of target samples (ESCC)	References
GSE26886	20 specimens of Barrett’s esophagus patients, 21 specimens of adenocarcinoma patients, 19 biopsies from patients with normal esophageal squamous epithelium, 9 specimens of squamous cell carcinoma	69	50	Wang et al. (2013) BMC Cancer
GSE69925	274 biopsy specimens in esophageal squamous cell carcinomas	274	274	Aoyagi et al. (2011) PLoS One
GSE32701	20 biopsy (BPY) and 20 surgical samples derived from the cancerous portion of the esophagus of 20 esophageal cancer patients	40	40	Aoyagi et al. (2011) PLoS One
GSE21293	35 genetically engineered human esophageal cells with different invasive abilities	35	35	Michaylira et al. (2010) Cancer Res

### Normalization

GC Robust Multi-array Average (GCRMA) algorithm including background correction, normalization, and summarization was performed to convert the CEL raw file to expression data which is based on R 3.5.1 script (Supplementary File S1). GCRMA normalization uses GC content of probes in normalization with RMA and gives one value for each probe set instead of keeping probe-level information. To run these analyses free *affy* (Gautier et al., 2004) and *gcrma* packages in R for the Affymetrix oligonucleotide array probe level data analysis, developed as part of the Bioconductor project, were downloaded (https://bioconductor.org/packages/affy/andhttps://bioconductor.org/packages/gcrma/). For each set of transcriptomes filtering and centering procedures were carried out using the Matlab R2019a (TheMathWorks, Inc.) development environment. Combining probsets corresponding to one gene was carried out according to the median value.

### Independent Component Analysis

In order to run the ICA, the BIODICA tool (ICA of BIg Omics Data), available athttps://github.com/LabBandSB/BIODICA was used. It provides both a command-line and a user-friendly Graphical User Interface for high-performance ICA analysis, including bootstrapping and further stability analysis. It allows the computation of the Maximally Stable Transcriptome Dimensionality (MSTD) index, which can be used to determine the optimal number of independent components (Kairov et al., 2017). ICA was applied to each transcriptomic dataset separately. For each analyzed transcriptomic dataset, 30 independent components (ICs) were computed.

### Comparison of Independent Components Across Datasets Using Correlation-Based Graphs

The similarity of two ICs obtained in different datasets was assessed by calculating the absolute value of Pearson’s correlation coefficient for the projection values of their common genes using corr () function in R. The highly correlated ICs with correlation coefficient r > 0.3 were selected and the results of the correlation computation across various datasets were summarized in a correlation graph**.** Construction and visualization of gene networks and graphs were performed using the Cytoscape v3.7.1 (http://www.cytoscape.org/) (Killcoyne et al., 2009), BiNoM plug-in (Zinovyev et al., 2008).

### PPI Network Construction

Cancer-associated protein-protein interactions networks were built by mapping highly expressed top 1,000 genes from each independent component into The Human Protein Reference Database (HPRD) (Prasad et al., 2009).

### Gene Set Enrichment Analysis (GSEA)

In order to identify the gene sets which are enriched in the list of selected (contributing) genes, computational software GSEA (http://software.broadinstitute.org/gsea/) implemented in BIODICA was used (Reimand et al., 2019). FDR threshold was set at 0.01 and *p*-value threshold at 0.01.

### ToppGene Analysis

Functional annotation-based candidate gene prioritization method for gene list enrichment analysis and candidate gene prioritization based on functional annotations and protein interactions network was performed using the interface to ToppGene web-service implemented in the BIODICA software (Chen et al., 2009).

**FIGURE 1:**
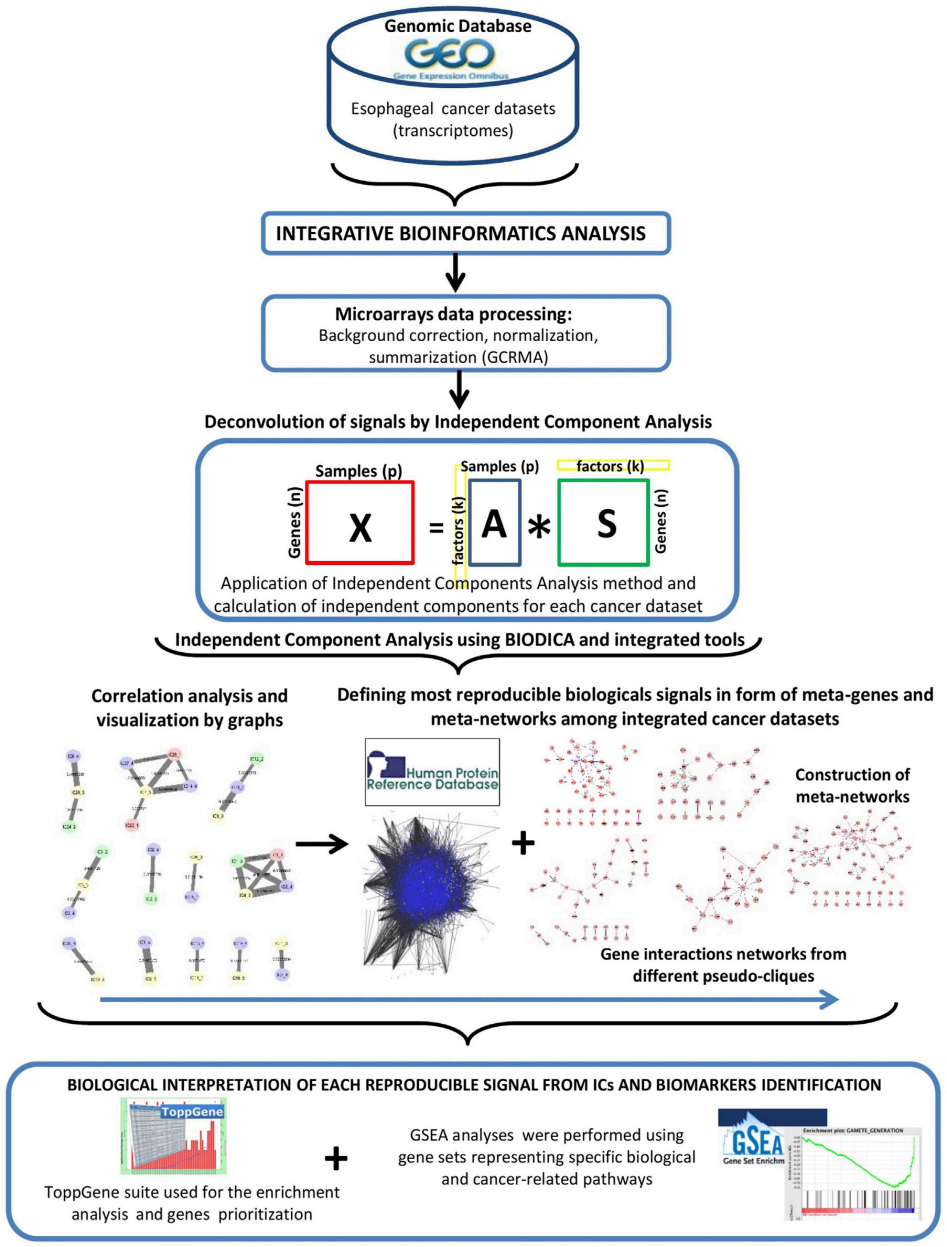
Schematic representation of the methodology.

Methodological workflow of this study is presented in Figure 1.

## Results

### Experimental Data

GSE26886 contains patient samples from 19 healthy subjects, 20 specimens from patients with Barrett’s esophagus, 21 cases of esophageal adenocarcinoma, and 9 cases of esophageal squamous cell carcinoma.

GSE69925 contains gene expression profiles of 274 biopsy specimens in esophageal squamous cell carcinomas.

GSE32701 contains gene expression profiles between 20 biopsies (BPY) and 20 surgical samples derived from the cancerous portion of the esophagus of 20 esophageal cancer patients. GSE21293 contains the mRNA profiles of 35 invasive and non-invasive genetically engineered human esophageal cell samples.

### Correlation Graph Between Independent Components

An analysis of the relationships between the calculated independent components from different sets of cancer transcriptomes was conducted by calculating Pearson’s correlation coefficients. On the basis of the values of the correlation coefficients (Supplementary File S2), undirected correlation graphs were obtained, reflecting the relationship between the IC (Supplementary File S3). The correlation graph is a connected structure in the form of pseudocliques, the nodes of which are correlated independent components. Each color corresponds to a specific cancer dataset: pink—GSE26886, green—GSE69925, yellow—GSE32701, blue—GSE21293. In the correlation graph with correlation coefficients R > 0.3 pseudocliques were observed, which are characterized by multiple relationships with independent components from different sets. The thickness of the edges between the nodes of the pseudoclique depends on the correlation coefficient (the larger the coefficient, the greater the thickness of the edges). The 12 pseudocliques were selected for constructing signal pathways for gene interaction.

### PPI Network Analysis

**FIGURE 2:**
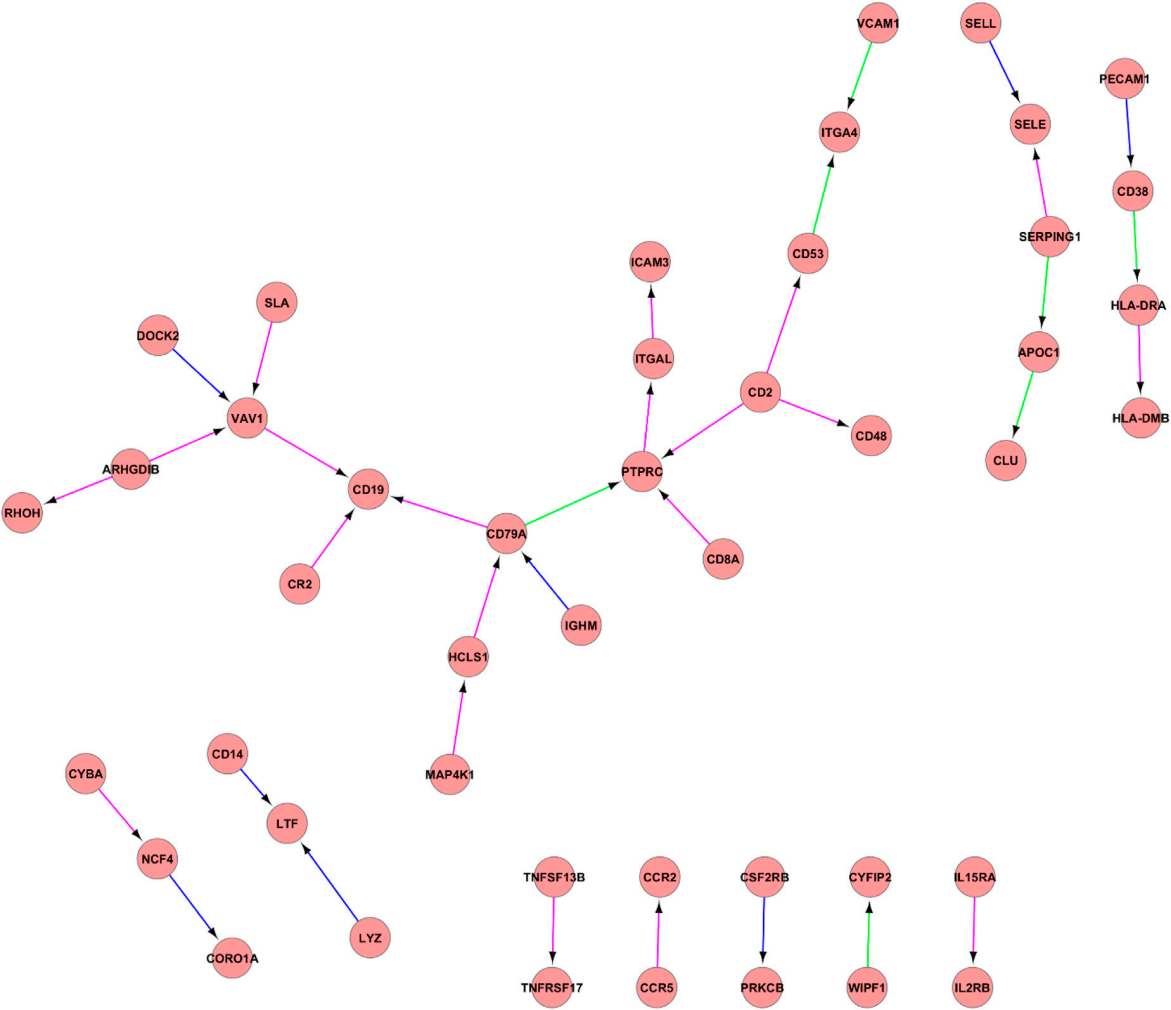
PPI network between IC3_2, IC3_3, and IC3_4. PPI network between IC3_2, IC3_3, and IC3_4. Proteins are illustrated with circles and directed interactions are illustrated with edges. Color of the edges represents the type of experiments used in HPRD database: blue—*in vitro*, red—*in vivo*, green—Y2H. This representation was obtained using Cytoscape software according to the HPRD database.

The analysis of constructed PPI networks using the IC3_2, IC3_3 and IC3_4 components (Figure 2). Identified pseudoclique consisted of a hub of genes with the central PTPRC gene network, CD2, CD19, CD14, CD48, CD53, CD8A, CD79A, CD38.

**FIGURE 3:**
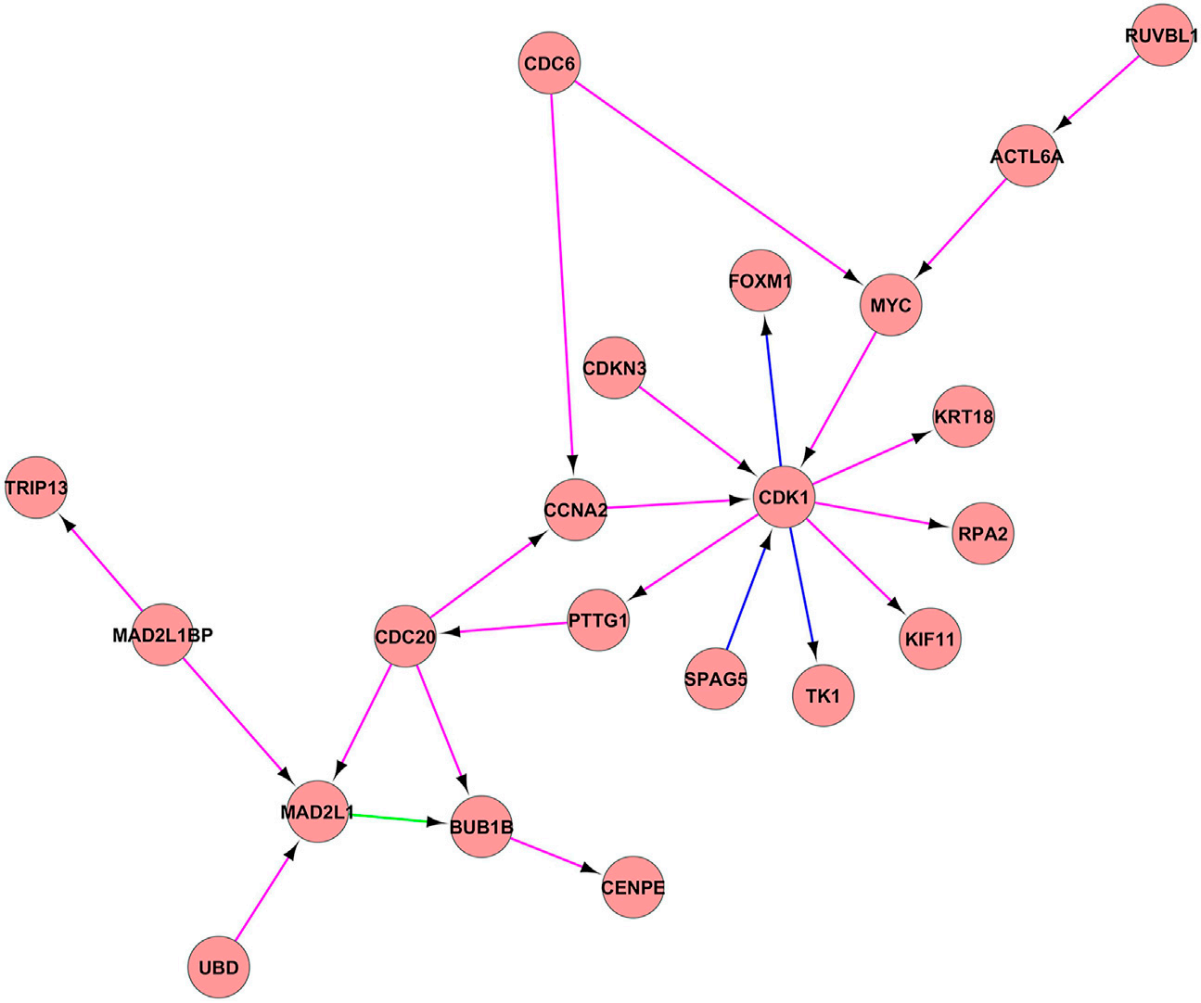
PPI network between the IC7_3 and IC14_4. PPI network between the IC7_3 and IC14_4. Proteins are illustrated with circles and directed interactions are illustrated with edges. Color of the edges represents the type of experiments used in HPRD database: blue—*in vitro*, red—*in vivo*, green—Y2H. This representation was obtained using Cytoscape software according to the HPRD database.

The analysis of constructed PPI network using the IC7_3 and IC14_4 showed the interaction of the following genes (Figure 3): CDK1, CCNA2, CDKN3, FOXM1, MYC, KRT18, RPA2, KIF11, TK1, SPAG5, PTTG1, CDC20, MAD2L1, MAD21BP, TRIP13, UBO, BUB1B, CENPE, CDC6, RUVBL1, ACTL6A all of these genes being known player of cell cycle machinery.

The analysis of constructed proteın-proteın network between the IC1_2 and IC8_4 revealed the interaction of the several proteins associated with Epithelial-mesenchymal transition (EMT) including keratin genes (KRT13, KRT6B, KRT6A, KRT19, KRT15) (Supplementary File S4).

The analysis of constructed proteın-proteın network between the IC20_3, IC24_2 and IC6_4 revealed the interaction of the several proteins associated with extracellular matrix genes (Supplementary File S5): FN1, DCN, FBLN2, FBLN5, FBN1, COL1A2, COL3A1, COL5A1, SPARC, MMP1, MMP7, APOC1, APOE, SPP1, CTSK, SERPING1, SFRP2, VCAN, BGN, A2M, LUM, CTSK, MFAP2, WISP1, DDR2, PDGFA, SPP1, IGDCC4, TGFBI.

The hub genes identified from this PPI network of IC5_4 and IC21_3 included STAT1 gene, C-C chemokine ligand family genes (CCl3, CCL4, CCL5, CCL3 like-1 (CCL3L1), CCL3 like-3 (CCL3L3)), C-C motif chemokine receptor family genes (CCR1, CCR5), MHC-I genes for human leukocyte antigens (HLA-C, -E, -F, -B), the β2 microglobulin (B2M) and genes of toll-like receptors (TLR3, TLR5, TLR8) (Supplementary File S6).

Results of GSEA and ToppGene analysis are summarized in Supplementary File S7.

## Discussion

Obtained results present the molecular pathways derived from four esophageal transcriptomic datasets. We focused on the deconvolution of gene expression profiles into independent components and combined those results with GSEA and ToppGene enrichment analysis.

Figure 2 reflects the gene interaction network of one of the identified pseudocliques composed of IC3_2, IC3_3 and IC3_4. In the central part of the network, there is one of the “hubs” of the PTPRC gene network—protein tyrosine phosphatase receptor type C, a factor for stimulating cell growth, differentiation, and oncogenic transformation. PTPRC, also known as CD45 antigen, has an important role in T and B cell activation and is associated with a poor prognosis of papillary thyroid carcinoma (Wu et al., 2019). Also, other clusters of differentiation or CD antigens (CD2, CD19, CD14, CD48, CD53, CD8A, CD79A, CD38) play important roles in the T and B cell receptor signaling pathways. In previous studies, the important roles of identified candidate biomarkers associated with immune processes of EC development were reported. For example, CD2 is responsible for the adhesion between T-cells and other cell types and T-cell activation, while CD8A identifies cytotoxic/suppressor T-cells that interact with MHC class I bearing targets. Furthermore, CD79A, also known as the B-cell antigen receptor complex, plays a functional role in the tumor-promoting effects of myeloid cells (Luger et al., 2013). It was identified that CD19 serves as B cell markers. All stages of B cell differentiation are accompanied by the expression of CD19 with the exception of final differentiation to plasma cells. In addition, the study of Li et al. showed that high expression of CD38 can indicate poor survival of ESCC patients (Li et al., 2017). Based on this gene network analysis, as well as the results of ToppGene (GO: Biological Process: humoral immune response, immune response-activating cell surface receptor signaling pathway) and GSEA (HALLMARK_COMPLEMENT, HALLMARK_INTERFERON_GAMMA_RESPONSE, HALLMARK_EPITHELIAL_MESENCHYMAL_TRANSITION), this pseudoclique can be interpreted as representing signals of immune-related response.

In Figure 3, PPI networks composed of IC7_3 and IC14_4 components were illustrated. The results of GSEA of this pseudoclique showed the enrichment of the gene sets involved in the cell cycle proliferation (HALLMARK_E2F_TARGETS, HALLMARK_G2M_CHECKPOINT, HALLMARK_MITOTIC_SPINDLE). Cell cycle progression has a critical role in cell proliferation, the deregulation of which has been identified as one of the cancer hallmarks. This checkpoint is responsible for the transition of the cell from the G2 phase to the M phase after DNA synthesis. As expected, the regulation of G2/M checkpoint by CDK1 gene (Cyclin-Dependent Kinase 1) was identified as an essential factor in multiple tumor progressions (Sedlacek et al., 1996; Damiens and Meijer, 2000; Knudsen and Witkiewicz, 2017; Chung et al., 2019). It also serves as the only CDK that can trigger the progression of mitosis (Santamaria et al., 2007). In addition, the study of Cyclin-dependent kinase inhibitor-3 (CDKN3) revealed that overexpression of CDKN3 promoted cell proliferation, migration, and invasion in esophageal squamous cell carcinoma (Liu J. et al., 2019). CDKN3 itself is a phosphatase, which acts as a tumor suppressor and mediates the cell cycle by dephosphorylation of cyclin-dependent kinases which mainly include CDK1. It was found that overexpression of CDKN3 in ESCC tissues could accelerate the proliferation of ESCC cells by accelerating G1/S transition, which suggested the oncogenic role of CDKN3 in human ESCC. Cell division cycle 20 homolog (CDC20) is also studied as one of the significant regulators of the cell cycle in multiple cancer types including breast cancer (Karra et al., 2014), prostate cancer (Zhang et al., 2019), and colorectal cancer (Wu et al., 2013). Also, studies have shown that overexpression of BUB1B (budding uninhibited by benzimidazole-related 1) was associated with progression of bladder cancer (Yamamoto et al., 2007), hepatocellular carcinoma (Liu et al., 2009), and prostate cancer (Fu et al., 2016). BUB1B is necessary for normal progression of mitosis, as its activity delays the initiation of anaphase-promoting complex/cyclosome (APC/C) by inhibiting the binding of CDC20 to APC/C. The other function of BUB1B is to regulate the kinetochore activities that depend on the kinetochore motor CENPE, which stands for Centromere-associated protein E. CENPE has an essential role in chromosome compression, microtubule-kinetochore conjugation, and spindle assembly checkpoint activation (Abrieu et al., 2000). While the Cell division cycle 6 homologue (CDC6) has been identified as an important regulator of DNA synthesis, and activator of checkpoints mechanisms (Deng et al., 2016). While the Kinesin family member 11 (KIF11) is mainly associated with the formation of bipolar mitotic spindles during cell division. There is evidence that KIF11 was overexpressed in the early stages of the breast (Jiang et al., 2017), colorectal, and ESCC (Imai et al., 2017). As the analysis showed the enrichment of the genes associated with the G2/M checkpoint and with the targets of E2F transcription factors, this clique can be associated with cell cycle proliferation.

The analysis of constructed proteın-proteın network between the IC1_2 and IC8_4 in Supplementary File S4 revealed the interaction of the several proteins associated with Epithelial-mesenchymal transition (EMT) including keratin genes (KRT13, KRT6B, KRT6A, KRT19, KRT15). Moreover, the enrichment of the following hallmark gene sets was identified: HALLMARK_EPITHELIAL_MESENCHYMAL_TRANSITION, HALLMARK_ESTROGEN_RESPONSE_LATE. EMT is a biological event in which epithelial cells lose their polarity and cell-cell adhesion and concomitantly acquire mesenchymal traits (Xiao et al., 2017). It has been reported that by combining two mesenchymal markers, vimentin and E-cadherin, EMT statues of many cancer types can be identified. For instance, in the study of Yamada et al. EMT stages of human pancreatic cancer was revealed and its correlation with lymph node metastasis was demonstrated (Yamada et al., 2013). According to the findings of Liu et al. it has been identified that tumor invasion, metastasis, and future prognosis of EC were also significantly correlated with EMT status (Liu et al., 2014). Thus, our study revealed other potential tumor-associated biomarker genes, namely keratin genes (KRTs), involved in EMT. The genes of the keratin family are responsible for the expression of various intermediate filament proteins found in epithelial tissues of various organs and they are differentially expressed in numerous human tumor malignancies as they are involved in tumor related processes such as invasion, metastasis, proliferation, and apoptosis of tumor cells (Zheng et al., 2021). Several studies showed the association of KRT13 with multiple cancer types including prostate, head and neck cancer, urothelial cancer (Li et al., 2016). Also, the KRT6A gene plays a critical role in epidermalization of squamous epithelium and in the EMT of nasopharyngeal carcinoma. Overexpression of KRT6A was associated with a poor prognosis of lung adenocarcinoma, as it promotes proliferation and metastasis of lung cancer via EMT and cancer stem cells transformation (Yang et al., 2020). Moreover, the KRT6B and KRT15 were reported as the markers of basal-like breast cancers (Charafe-Jauffret et al., 2006). In addition, KRT19, which is responsible for maintaining the structural integrity of epithelial cells, was reported as tumor-associated proteins as its down-expression was identified in ESCC in a proteomic study. While the KRT13 was overexpressed in ESCC tissue compared to adjacent normal tissues (Zhang et al., 2011). The results of He et al. revealed that KRT13 was responsible for the cell cycle arrest and inhibition of growth in response to the EC (He et al., 2015). By analysis of this clique, it can be concluded that genes of these components are involved in the induction of the EMT process of cancer metastatic progression.

Correlation analysis of network between IC20_3, IC24_2 and IC6_4 in Supplementary File S5 showed the interaction of following genes: FN1, DCN, FBLN2, FBLN5, FBN1, COL1A2, COL3A1, COL5A1, SPARC, MMP1, MMP7, APOC1, APOE, SPP1, CTSK, SERPING1, SFRP2, VCAN, BGN, A2M, LUM, CTSK, MFAP2, WISP1, DDR2, PDGFA, SPP1, IGDCC4, TGFBI. The results of GSEA of this clique showed the enrichment of the gene sets involved in extracellular matrix (ECM)-receptor interaction (HALLMARK_INTERFERON_GAMMA_RESPONSE, HALLMARK_EPITHELIAL_MESENCHYMAL_TRANSITION, HALLMARK_TNFA_SIGNALING_VIA_NFKB). COL1A2, COL3A1, COL5A1 encode the collagen family, whose members are the main components of the tumor-stromal environment and play an important role in behavior of cancer cells. Collagen III (COL3A1 gene) forms reticular fibers that keep the ECM together. The dysregulation of COL1A2 (Collagen, type I, alpha 2) and its receptor DDR2 (Discoidin domain receptor 2) can cause various collagen-associated effects in tumors. The study investigating the association between expression of COL1A2 and EC revealed the abnormal expression of this collagen gene in EC tissues. Moreover, COL1A2 served as a direct target gene of miR-133a-3p and showed inhibition of its function by promotion of apoptosis of ECSS (Li et al., 2020a). FN1 (Fibronectin 1) is a heterodimeric form of glycoprotein on the surface cells and participates in the adhesion of cells to the ECM, as well as in the processes of cell migration, wound healing, blood clotting and immune response. Authors of a recent study found that FN1 was associated with tumorigenesis of esophageal carcinoma as its overexpression correlated with a higher pathological stage of EC (Li et al., 2020b). TGFBI (transforming growth factor-beta-induced protein) is secretory extracellular matrix protein induced by TGF-β. Cell adhesion of ECM proteins including collagen, fibronectin, and laminin is mediated by TGFBI and its function was widely investigated in various tumor progression. Ozawa et al. have reported that overexpression of TGFBI was associated with poor prognosis in ESCC samples (Ozawa et al., 2016). Matrix metalloproteinases (MMPs) have been reported to be a crucial factor during tumor invasion and metastasis through degradation of ECM compartments. For instance, MMP-7 degrades various components of basement membrane such as laminin and specific collagens as well as it activates other MMP family members including MMP1 (Yamashita et al., 2000). Authors of another study also have reported that MMP7 expression via transcription factor activin A correlates with the aggressiveness of EC (Yoshinaga et al., 2008). Finally, these analyses reported the overexpression of MMP7 as a significant prognostic marker of EC. VCAN, gene that encodes stromal protein of cancer-associated fibroblasts—versican, is an important component of ECM due to its function in inflammation and immunity during progression of various cancers including breast, gastric cancers and leukemia (Wight et al., 2020). The recent findings indicated that stromal overexpression of versican can serve as prognostic biomarker of ESCC (Yamauchi et al., 2021). According to the analysis of this PPI network, it was reported that the most commonly enriched function of these genes was ECM-receptor interaction. As the proteins of ECM such as collagens, fibronectin, matrix metalloproteinases play crucial role in tumor invasion and metastasis, comprehensive understanding of ECM-cell interaction and its underlying mechanism in tumor initiation and progression would contribute to the development of potential biomarkers and therapeutic targets for EC treatment.

According to the GSEA results of identified PPI network between the IC5_4 and IC21_3 in Supplementary File S6, several pathways were related to immune processes (HALLMARK_INTERFERON_ALPHA_RESPONSE, HALLMARK_INFLAMMATORY_RESPONSE, HALLMARK_COMPLEMENT), developmental process (HALLMARK_EPITHELIAL_MESENCHYMAL_TRANSITION) as well as to signaling process (HALLMARK_KRAS_SIGNALING_UP).

The hub genes identified from this PPI network included STAT1 gene, C-C chemokine ligand family genes (CCl3, CCL4, CCL5, CCL3 like-1 (CCL3L1), CCL3 like-3 (CCL3L3)) and C-C motif chemokine receptor family genes (CCR1, CCR5). STAT1 (signal transducer and activator of transcription) gene is reported as a tumor suppressor in ESCC (Liu et al., 2018). CCL3, also known as macrophage inflammatory protein-1α (MIP-1α), is a ligand for CC chemokine receptor 1 (CCR1) and CC chemokine receptor 5 (CCR5). It was identified that CCL3 and CCL5 have been associated with the progression of various malignancies (Aldinucci and Colombatti, 2014). For instance, CCL3–CCR5 axis lead to the process of osteolysis in multiple myeloma, lung metastasis in murine renal cell carcinoma, angiogenesis in osteosarcoma, whereas the CCL3–CCR1 axis is also involved in the progression of hepatocellular carcinoma. Moreover, CCL3–CCR5 axis appear to be involved in the progression of ESCC by activating Akt and ERK signaling pathways and by promoting the migration and invasion of cancer cells and angiogenesis (Kodama et al., 2020). In addition, both of these axes are involved in leukemogenesis of chronic myeloid leukemia and in the progression of oral squamous cell carcinoma. Another cluster of genes consists of MHC-I genes for human leukocyte antigens (HLA-C, -E, -F, -B) and the β2 microglobulin (B2M). It was found that HLA-F was associated with several HLA complexes including HLA-B, HLA-C, and HLA-E. Furthermore, HLA-B and HLA-C mainly present antigens to CD8T cells and participate in the regulation of several immunologic functions. Whereas, HLA-E and HLA-F found to be involved in the regulation of NK cell function through its receptor (Dulberger et al., 2017). In esophageal squamous cell carcinoma, the expression of HLA-F was significantly correlated with the poor survival in patients (Zhang et al., 2013). One of the networks includes genes encoding Toll-like receptors (TLR3, TLR5, TLR8) and myeloid differentiation primary response-88 (MyD88) adaptor protein which has been identified to mediate inflammation (Zheng et al., 2020). A variety of TLRs such as TLR3 and TLR5 have been shown to be overexpressed in esophageal squamous cell carcinoma and esophageal adenocarcinoma (Kauppila and Selander, 2014). The major interactions of this network show the association with immune related processes such as antigen processing and presentation, immune response and inflammatory response.

In conclusion, our study aimed to propose an investigative approach for meta-analysis of esophageal cancer transcriptomes with implementation of matrix factorization method. Our implemented approach of applying ICA and deconvolution of signals from gene expression profiles revealed several molecular pathways enriched in EC. Comprehensive analysis including GSEA, toppGene and PPI network analysis provided the significant correlation between immune-related genes, EMT-associated genes, ECM-receptor interaction genes, and cell cycle-related biomarkers with the development of EC. These findings may reveal esophageal cancer-related genomic signatures that can be used as predictive biomarkers and potential targets for early diagnosis and antitumor therapies. However, since our findings were based only on the meta-analysis of independent esophageal cancer transcriptomes, further experimental studies on these identified pathways and genes are still needed. Also, integration of different multilevel « multi-omics » datasets with the systematic application of ICA may improve the methodology and reveal additional non-transcriptomics biomarkers associated with esophageal cancer.

## Data Availability

The original contributions presented in the study are included in the article/Supplementary Material, further inquiries can be directed to the corresponding author.
