# Supplementary material for: Meta-Analysis of Esophageal Cancer Transcriptomes Using Independent Component Analysis
Source: Front Genet. 2021 Oct 21;12:683632. doi: 10.3389/fgene.2021.683632 (PMC8594933; doi:10.3389/fgene.2021.683632)
Supplement: Supplementary file 1 [file DataSheet1.docx]

***Supplementary Material***

**Supplementary file S1 – R Script.**

# Affymetrix

library(affy)

library(hgu133plus2cdf)

celfiles <- list.files("raw", full.names=TRUE)

affy.raw <- ReadAffy(filenames = celfiles)

print("CEL files loaded")

print(affy.raw)

##QC - Affy

library(simpleaffy)

library(annotation)

library(arrayQualityMetrics)

affy.raw <- ReadAffy(filenames = celfiles)

affy.qc <- qc(affy.raw)

plot(affy.qc)

library(arrayQualityMetrics)

affy.raw <- ReadAffy(filenames = celfiles)

arrayQualityMetrics(affy.raw, outdir = "qc")

##this wrote pdf report to "qc" directory

##CDF

affy.raw@cdfName <- "HG-U133_Plus_2"

library(simpleaffy)

library(annotation)

library(arrayQualityMetrics)

affy.qc.data <- ReadAffy(filenames = celfiles)

pdf(file=qc.file)

affy.qc <- qc(affy.qc.data)

plot(affy.qc)

qc.file <- "affy_qc.pdf"

dev.off()

##GCRMA Normalization

library(gcrma)

affy.data <- gcrma(affy.raw)

print("GCRMA summarization finished")

print(affy.data)

print(head(exprs(affy.data)))

write.exprs(affy.data, "affy_gcrma.txt")

**Supplementary file S2 – Correlation values between ICs (R>0.3).**

| **IC interactions** | **Correlation Value** |
| --- | --- |
| IC22_1__IC7_3 | 0.303792 |
| IC26_3__IC25_4 | 0.31731156 |
| IC12_2__IC23_4 | 0.30496705 |
| IC2_4__IC2_2 | 0.35446236 |
| IC2_4__IC1_3 | 0.5461127 |
| IC29_1__IC27_4 | 0.3267412 |
| IC8_4__IC4_3 | 0.52057457 |
| IC2_3__IC1_4 | 0.54186165 |
| IC3_3__IC3_4 | 0.30217952 |
| IC7_3__IC29_1 | 0.42810875 |
| IC14_4__IC29_1 | 0.30996543 |
| IC7_3__IC14_4 | 0.45044604 |
| IC10_3__IC30_4 | 0.37320092 |
| IC11_3__IC13_4 | 0.3271076 |
| IC19_4__IC16_3 | 0.32361117 |
| IC23_4__IC9_3 | 0.45640343 |
| IC1_1__IC4_3 | 0.3711783 |
| IC1_1__IC8_4 | 0.35808846 |
| IC1_2__IC1_1 | 0.38636208 |
| IC1_2__IC4_3 | 0.46617225 |
| IC1_2__IC8_4 | 0.4279809 |
| IC27_4__IC7_3 | 0.35242233 |
| IC20_3__IC24_2 | 0.36360705 |
| IC20_3__IC6_4 | 0.45053947 |
| IC3_2__IC3_3 | 0.4241125 |
| IC21_3__IC5_4 | 0.36203238 |

**Supplementary file S3 – Correlation graph**


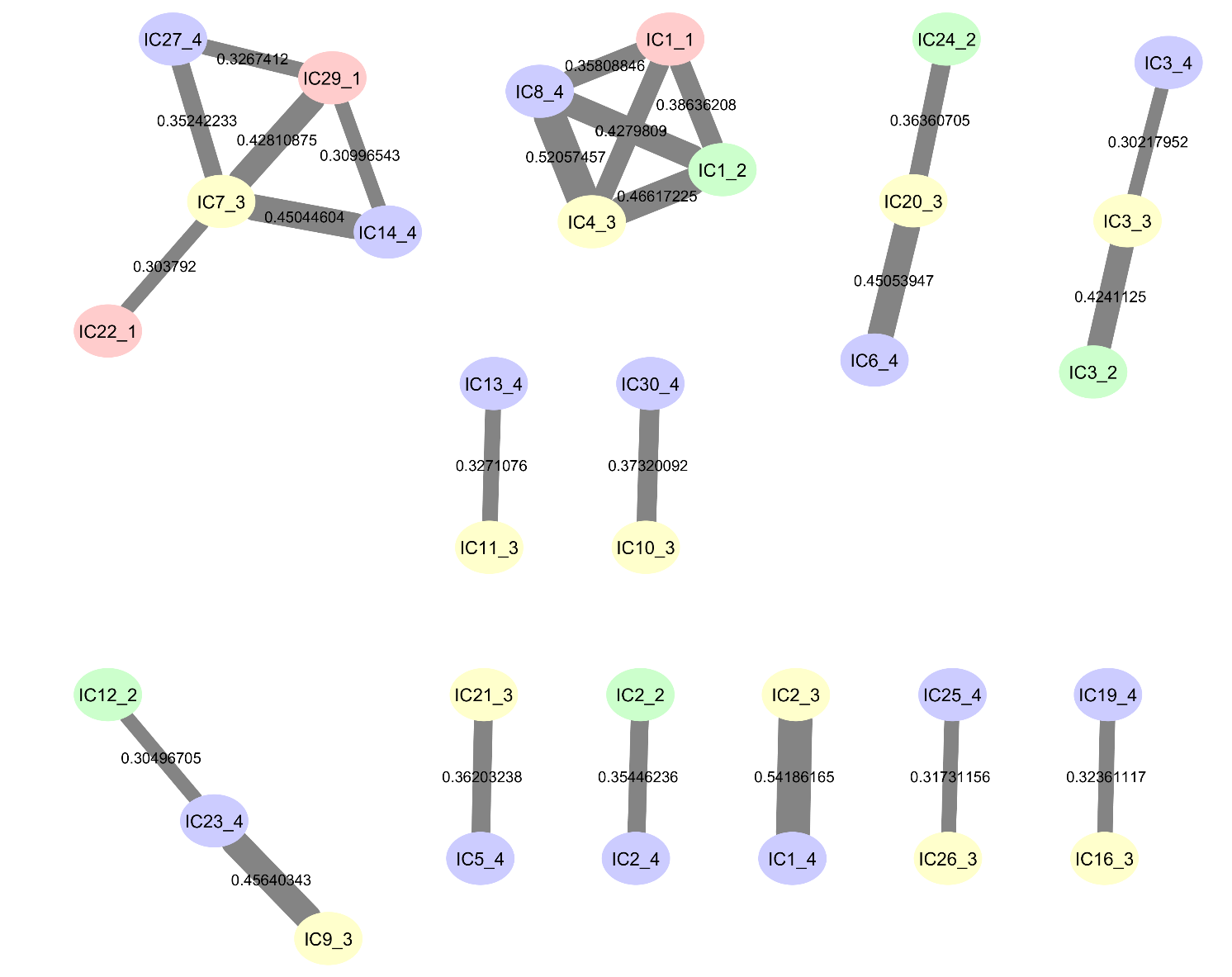


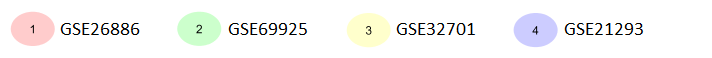


**The correlation graph between decompositions into 30 ICs was built with absolute correlation values exceeding 0.3.**

The correlation graph is a connected structure in the form of clicks or pseudo-clicks, the nodes of which are correlated independent components. Each color corresponds to a specific cancer dataset: pink – GSE26886, green – GSE69925, yellow – GSE32701, blue – GSE21293. In the correlation graph with correlation coefficients R>0.3 pseudo-clips were observed, which are characterized by multiple relationships with independent components from different sets. The thickness of the edges between the nodes of the clique depends on the correlation coefficient (the larger the coefficient, the greater the thickness of the edges). The 12 pseudo-clicks were selected for constructing signal pathways for gene interaction.

**Supplementary file S4. PPI network between the IC1_2 and IC8_4**
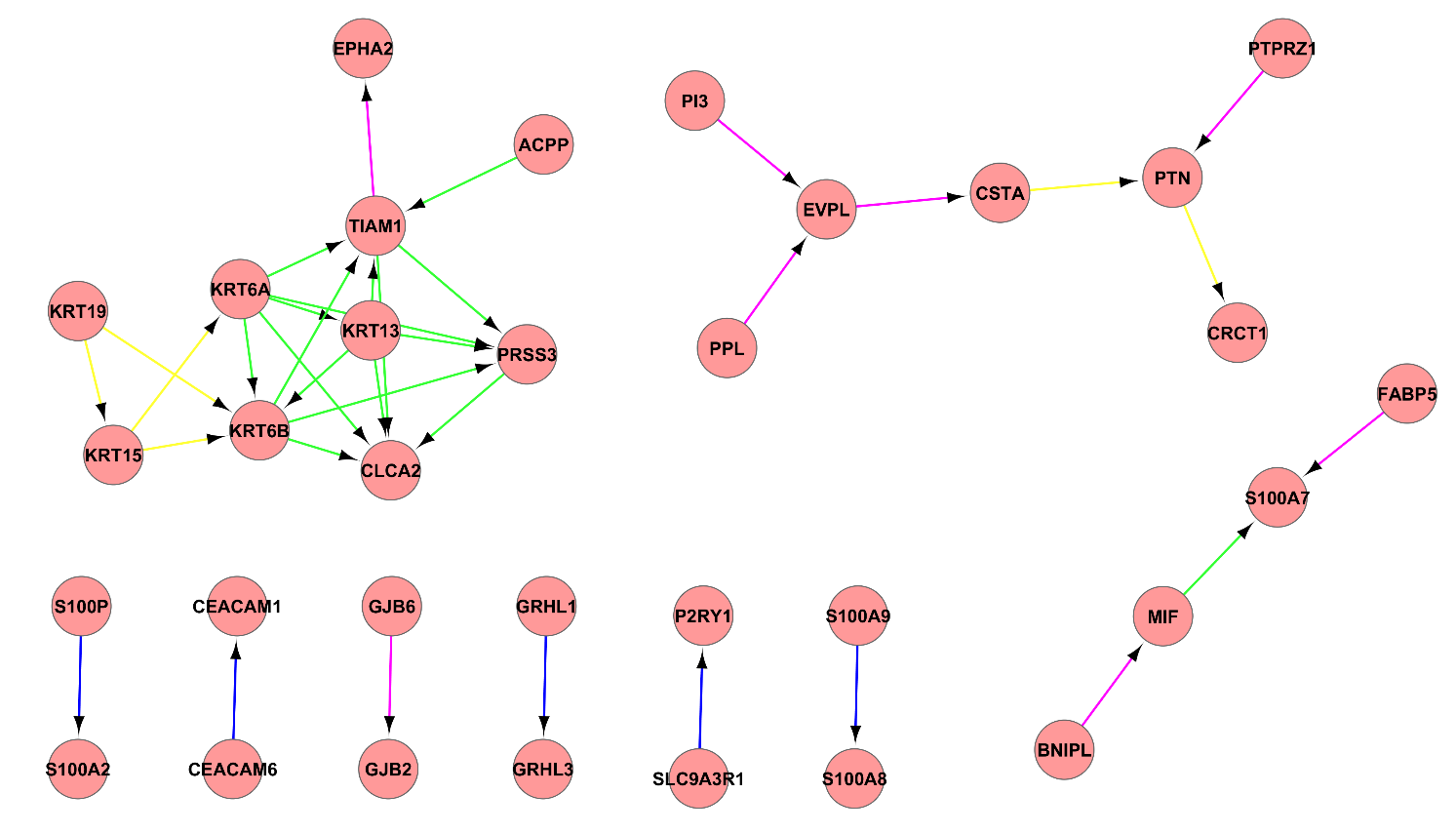


**PPI network between the IC1_2 and IC8_4.** Proteins are illustrated with circles and directed interactions are illustrated with edges. Color of the edges represents the type of experiments used in HPRD database: blue - in vitro, red – in vivo, green – Y2H. This representation was obtained using Cytoscape software according to the HPRD database.

**Supplementary file S5. PPI network between the IC20_3, IC24_2 and IC6_4**

**
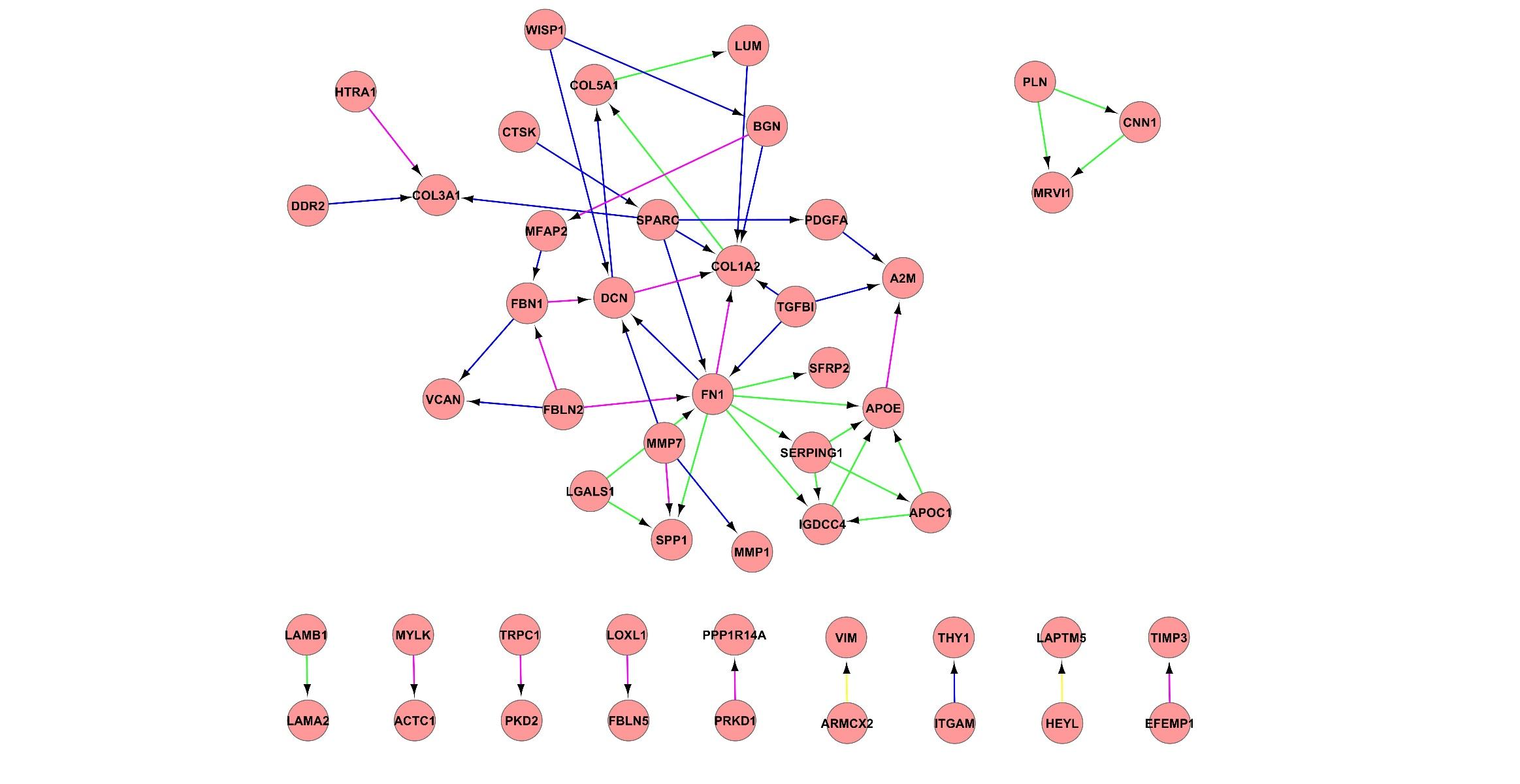
**

**PPI network between the IC20_3, IC24_2, and IC6_4.** Proteins are illustrated with circles and directed interactions are illustrated with edges. Color of the edges represents the type of experiments used in HPRD database: blue - in vitro, red – in vivo, green – Y2H. This representation was obtained using Cytoscape software according to the HPRD database.

**Supplementary file S6. PPI network between the IC5_4 and IC21_3**

**
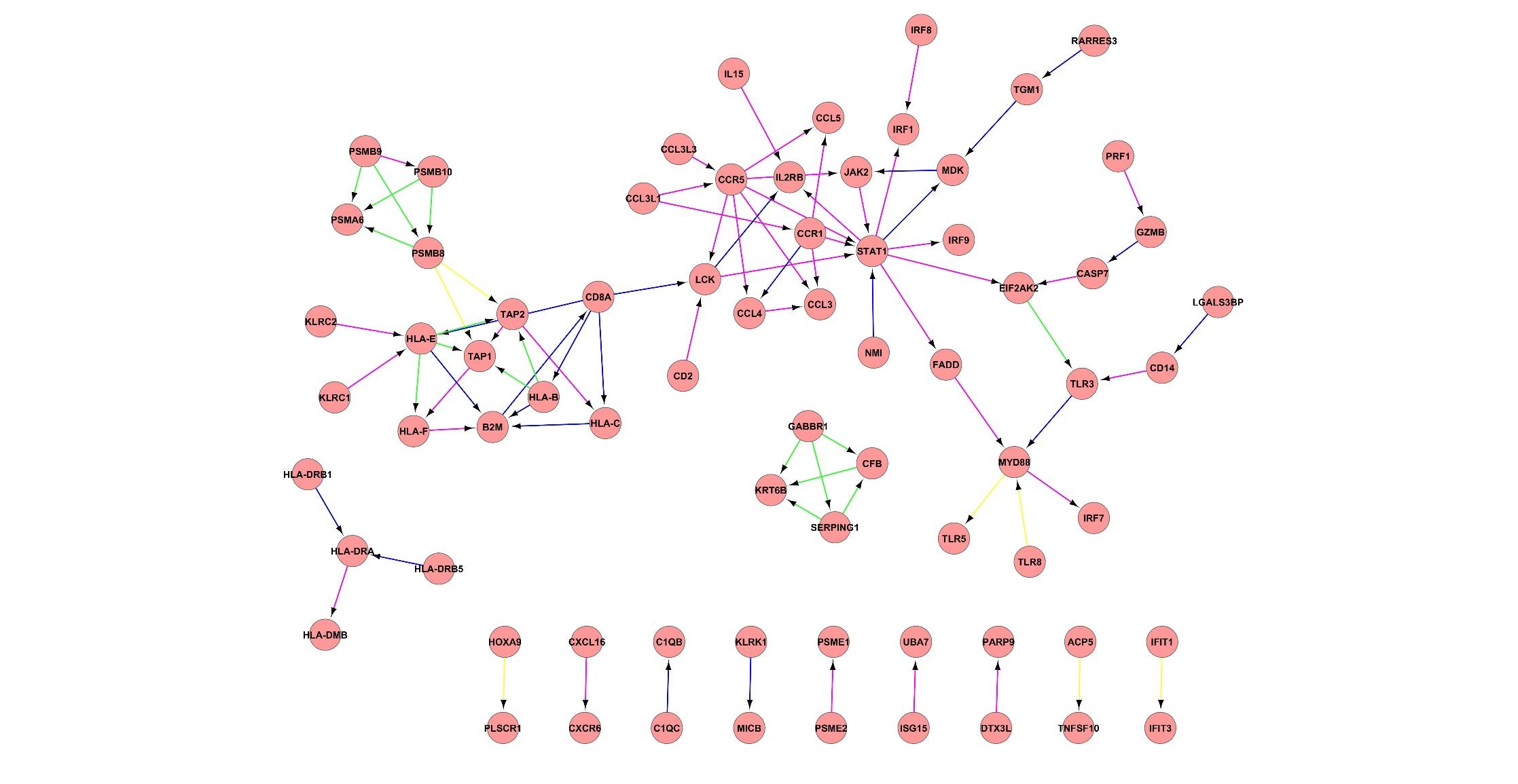
**

**PPI network between the IC5_4 and IC21_3.** Proteins are illustrated with circles and directed interactions are illustrated with edges. Color of the edges represents the type of experiments used in HPRD database: blue - in vitro, red – in vivo, green – Y2H. This representation was obtained using Cytoscape software according to the HPRD database.

**Supplementary file S7. GSEA and ToppGene analysis results**

| Clique | Components | GSEA | ToppGene | | |
| --- | --- | --- | --- | --- | --- |
|  |  |  | BP | MF | CC |
| 1 | IC3_2, IC3_3, and IC3_4 | HALLMARK_COMPLEMENT, HALLMARK_INTERFERON_GAMMA_RESPONSE, HALLMARK_EPITHELIAL_MESENCHYMAL_TRANSITION | [humoral immune response](http://www.ebi.ac.uk/ego/DisplayGoTerm?id=GO:0006959), [immune response-activating cell surface receptor signaling pathway](http://www.ebi.ac.uk/ego/DisplayGoTerm?id=GO:0002429), cornified envelope assembly | [Immunoglobulin receptor binding](http://www.ebi.ac.uk/ego/DisplayGoTerm?id=GO:0034987), [antigen binding](http://www.ebi.ac.uk/ego/DisplayGoTerm?id=GO:0003823), [protein-glutamine gamma-glutamyltransferase activity](http://www.ebi.ac.uk/ego/DisplayGoTerm?id=GO:0003810) | [immunoglobulin complex](http://www.ebi.ac.uk/ego/DisplayGoTerm?id=GO:0019814), [cornified envelope](http://www.ebi.ac.uk/ego/DisplayGoTerm?id=GO:0001533) |
| 2 | IC7_3 and IC14_4 | HALLMARK_G2M_CHECKPOINT,  HALLMARK_MITOTIC_SPINDLE | [chromosome segregation](http://www.ebi.ac.uk/ego/DisplayGoTerm?id=GO:0007059) | [receptor ligand activity](http://www.ebi.ac.uk/ego/DisplayGoTerm?id=GO:0048018) | [mitochondrial protein complex](http://www.ebi.ac.uk/ego/DisplayGoTerm?id=GO:0098798), [neutrophil migration](http://www.ebi.ac.uk/ego/DisplayGoTerm?id=GO:1990266) |
| 3 | IC1_2 and IC8_4 | HALLMARK_EPITHELIAL_MESENCHYMAL_TRANSITION,  HALLMARK_ESTROGEN_RESPONSE_LATE | [snRNA 3'-end processing](http://www.ebi.ac.uk/ego/DisplayGoTerm?id=GO:0034472) |  | [integrator complex](http://www.ebi.ac.uk/ego/DisplayGoTerm?id=GO:0032039) |
| 4 | IC20_3, IC24_2 and IC6_4 | HALLMARK_INTERFERON_GAMMA_RESPONSE, HALLMARK_EPITHELIAL_MESENCHYMAL_TRANSITION, HALLMARK_TNFA_SIGNALING_VIA_NFKB | [extracellular matrix organization](http://www.ebi.ac.uk/ego/DisplayGoTerm?id=GO:0030198), [defense response to virus](http://www.ebi.ac.uk/ego/DisplayGoTerm?id=GO:0051607) | [extracellular matrix structural constituent](http://www.ebi.ac.uk/ego/DisplayGoTerm?id=GO:0005201) | [collagen-containing extracellular matrix](http://www.ebi.ac.uk/ego/DisplayGoTerm?id=GO:0062023) |
| 5 | IC5_4 and IC21_3 | HALLMARK_INTERFERON_ALPHA_RESPONSE,  HALLMARK_INFLAMMATORY_RESPONSE, HALLMARK_COMPLEMEN, HALLMARK_EPITHELIAL_MESENCHYMAL_TRANSITION, HALLMARK_KRAS_SIGNALING_UP | [type I interferon signaling pathway](http://www.ebi.ac.uk/ego/DisplayGoTerm?id=GO:0060337), [defense response to virus](http://www.ebi.ac.uk/ego/DisplayGoTerm?id=GO:0051607) | [chemokine receptor binding](http://www.ebi.ac.uk/ego/DisplayGoTerm?id=GO:0042379) | [MHC protein complex](http://www.ebi.ac.uk/ego/DisplayGoTerm?id=GO:0042611) |
